# The Bidirectional Interaction Between NF‐*κ*B and Glucocorticoid Receptor: Underlying Mechanisms of Chronic Stress‐Induced Pathology

**DOI:** 10.1155/jimr/5517840

**Published:** 2025-12-16

**Authors:** Hyun-June Yu, Hi-Joon Park, Bombi Lee, Dae-Hyun Hahm

**Affiliations:** ^1^ Department of Medicine, College of Medicine, Kyung Hee University, Seoul, 02447, Republic of Korea, khu.ac.kr; ^2^ Acupuncture and Meridian Science Research Center, Kyung Hee University, Seoul, 02447, Republic of Korea, khu.ac.kr; ^3^ Center for Converging Humanities, Kyung Hee University, Seoul, 02447, Republic of Korea, khu.ac.kr; ^4^ Department of Physiology, College of Medicine, Kyung Hee University, Seoul, 02447, Republic of Korea, khu.ac.kr

**Keywords:** cancer, chronic stress, glucocorticoid, glucocorticoid receptor, inflammation, nuclear factor-kappa b

## Abstract

Chronic stress is an established etiological factor for numerous pathologies, including cancer, yet the underlying molecular etiology remains incompletely understood. This review elucidates a critical molecular axis through which chronic stress promotes carcinogenesis via the bidirectional interaction between the glucocorticoid receptor (GR) and nuclear factor‐*κ*B (NF‐*κ*B). The review comprehensively details how chronic stress induces pathological GR signaling, characterized by post‐translational modifications (PTMs), glucocorticoid (GC) resistance, and altered expression of receptor isoforms. This impairment of GR function leads to the disinhibition of proinflammatory transcription factor, NF‐*κ*B. This disinhibition results in sustained NF‐*κ*B hyperactivation, which orchestrates a protumorigenic microenvironment by driving genetic instability, immune evasion, uncontrolled proliferation, apoptosis resistance, angiogenesis, and metastasis. By providing an integrative synthesis of these interconnected pathways, this review offers a novel mechanistic framework that directly links the molecular consequences of chronic stress to the hallmarks of cancer. This work therefore establishes the GR/NF‐*κ*B signaling interface as a critical and therapeutically targetable mediator of stress‐induced carcinogenesis.

## 1. Introduction

Chronic stress is a sustained state of psychological or physiological tension resulting from repeated exposure to stressors without adequate recovery. Over time, the persistent activation of the stress‐response system disrupts homeostasis, increasing vulnerability to a range of chronic diseases [1]. Epidemiological, clinical, and preclinical evidence increasingly implicated chronic stress as a contributor to cancer initiation, progression, and poorer outcomes [2].

However, much of the literature framed this link through a predominantly hormonal lens, focusing on the dysregulation of the hypothalamic–pituitaryadrenal (HPA) axis, aberrant glucocorticoid (GC) signaling, and neuroendocrine imbalances [3]. Such hormonal explanations, while valuable, fall short of fully accounting for the complexity of oncogenesis under stress. Concurrently, nuclear factor‐*κ*B (NF‐*κ*B) has emerged as a central transcriptional hub in inflammation‐associated cancers, facilitating proliferation, survival, angiogenesis, immune evasion, and metastasis [4]. Moreover, mounting evidence suggests that glucocorticoid receptors (GRs) interact dynamically with NF‐*κ*B signaling, enabling crosstalk between the endocrine stress response and tumor‐relevant transcriptional regulation [5].

**Figure 1:**
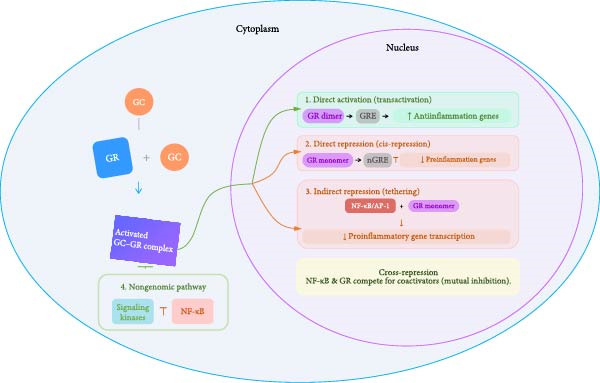
Proposed diagram of the stress response pathway in which GR and NF‐*κ*B play key roles. Changes in GC hormone levels and GR‐mediated anti‐inflammation processes are shown. Important flows are shown and detailed processes are excluded for concise display. ↑ = activation, increase, ↓ = inactivation, decrease, HPA = hypothalamic–pituitary‐adrenal, GC = glucocorticoid, GR = glucocorticoid receptor, CBG = cortisol‐binding‐globulin, GRE = glucocorticoid receptor element, NF‐*κ*B = nuclear factor kappa‐light‐chain‐enhancer of activated B cells.

To this date, comprehensive reviews that integrate how chronic stress alters GC sensitivity, modulates GR‐NF‐*κ*B crosstalk, and ultimately fosters carcinogenesis remain scarce (Figure 1). To address this gap, our review synthesizes extant literature to (1) examine theoretical and empirical evidence linking chronic stress to disruptions in GR and NF‐*κ*B associated pathways; (2) characterized GR modifications (including posttranslational changes and epigenetic regulation) and mechanisms underlying GR resistance under chronic stress; and (3) elucidate how the altered GR‐NF‐*κ*B axis can promote tumor initiation, progression, and resistance to therapy. By reframing chronic stress as a molecular integrator, rather than merely a hormonal disturbance, this review aims to advance our mechanistic understanding and identify actionable nodes within the GR‐NF‐*κ*B signaling network for future translational research.

## 2. Stress Response

### 2.1. The Sympathetic‐Adrenal‐Medullary (SAM) and Hypothalamic–Pituitary–Adrenal Axes

The stress response is primarily mediated by two systems: the SAM for immediate reactions and the HPA for sustained responses [6]. The SAM system reacts quickly to sudden stress, generating the “fight or flight” response as follows: the hypothalamus activates the sympathetic nervous system, which stimulates the adrenal medulla to release epinephrine (EPI) and norepinephrine (NE) [6]. These hormones increase blood pressure and heart rate and rapidly mobilize energy, enabling the body to respond immediately to a threat. In contrast, the HPA system is responsible for a slower and more prolonged hormonal response [7]. The hypothalamus secretes corticotropin‐releasing hormone (CRH), which acts on receptors (primarily CRH‐R1 in the brain) to stimulate the pituitary gland’s release of adrenocorticotropic hormone (ACTH) [7]. The activity of CRH is also tightly regulated by CRH‐binding protein (CRH‐BP), which sequesters CRH to prevent excessive activation [8]. Ultimately, ACTH triggers the adrenal cortex to produce the stress hormone cortisol. Cortisol binds to GR present in nearly all cells to regulate gene expression, exerting widespread effects, particularly on the immune system. The HPA axis is self‐regulated through a negative feedback loop, wherein elevated cortisol levels inhibit the hypothalamus and pituitary gland [9].

### 2.2. GR*α*: Mechanisms of Action and Immunomodulatory Role

GR*α* is a member of the nuclear receptor superfamily that functions as a ligand‐activated transcription factor. As a modular protein, it contains three primary functional domains: a largely unstructured N‐terminal domain (NTD), which harbors the main transactivation function (AF‐1), a central DNA‐binding domain (DBD) with two zinc fingers, and a C‐terminal ligand‐binding domain (LBD), which contains a ligand‐dependent activation function (AF‐2) [10]. In its inactive state, GR*α* resides in the cytoplasm as part of a large multiprotein complex with chaperone proteins such as heat‐shock protein 90 (hsp 90) and hsp 70. While 80% of circulating GCs are bound to cortisol‐binding globulin (CBG) and 10% to albumin, the remaining 10% are free and biologically active enough to diffuse across the cell membrane [11]. The transcriptional process begins when the GC ligand binds to the LBD of the GR*α*. This binding induces a conformational change, causing the dissociation of the chaperones and the formation of an “activated GR*α*” complex. The activated GR*α* complex then translocates into the nucleus, aided by proteins such as immunophilins (including FKBP4 and FKBP5) [11].

Once inside the nucleus, the activated GR*α* dimerizes and modulates gene expression through several key genomic and non‐genomic mechanisms:

#### 2.2.1. Direct Gene Activation (transactivation)

The primary mechanism of GR*α* action involves the direct binding of a GR*α* dimer to glucocorticoid response elements (GREs) located in the promoter regions of target genes [12]. This sequence‐specific binding is governed by the GR*α*’s DBD, which recruits coactivators and the basal transcription machinery, leading to the upregulation of gene expression. This transactivation mechanism is fundamental to the anti‐inflammatory effects of GCs, as it drives the expression of key antiinflammatory and pro‐resolution genes, including glucocorticoid‐induced leucine zipper (GILZ), mitogen‐activated protein kinase phosphatase‐1 (MKP‐1/DUSP1), and the NF‐*κ*B inhibitor, I*κ*B*α* (NFKBIA) [13, 14].

#### 2.2.2. Direct Gene Repression (cis‐repression)

The GR*α* dimer can also bind to negative GREs (nGREs) to directly suppress gene transcription [15]. This binding leads to the recruitment of corepressors, which interfere with the basal transcriptional complex. This mechanism is responsible for the downregulation of genes such as pro‐opiomelanocortin (POMC) in the HPA axis [16]. Moreover, thymic stromal lymphopoietin (TSLP), a critical cytokine in allergic inflammation, is also a direct target of this repressive mechanism through a specific nGRE sequence identified in its gene promoter [17]. This element’s unique sequence architecture structurally precludes GR*α* dimerization, enforcing a monomeric binding conformation that provides a definitive structural basis for direct gene suppression.

#### 2.2.3. Indirect Gene Repression (trans‐repression via tethering) and cross‐repression

A crucial component of the GR*α*’s antiinflammatory action does not involve direct DNA binding. Instead, an activated GR*α* can physically interact with, or tether to, other DNA‐bound proinflammatory transcription factors, such as NF‐*κ*B, AP‐1, cAMP response element‐binding protein (CREB), and signal transduction and activator of transcription (STAT) [15]. This protein–protein interaction, such as between GR*α* and the p65 subunit of NF‐*κ*B, prevents these factors from accessing target promoters or recruiting essential coactivators (such as CBP/p300), thereby halting the transcription of proinflammatory genes, including cytokines (IL‐1*β*, IL‐6, TNF‐*α*, and INF‐*γ*), chemokines, and adhesion molecules [18]. Conversely, this antagonism is reciprocal. Inflammatory transcription factors, particularly NF‐*κ*B, can also suppress the GR*α*’s ability to activate genes through cross‐repression. This occurs through coactivator competition, sequestering shared cofactors like CBP/p300 or via steric hindrance that prevents GR*α* from binding to GREs. This bidirectional repression maintains the balance between proinflammatory and antiinflammatory activity [19].

#### 2.2.4. Nongenomic Mechanisms

Beyond its classical genomic mechanisms, the GR exerts rapid nongenomic effects that are integral to immune regulation. Upon GC ligand binding, the cytosolic GR*α* directly interacts with signaling kinases such as Src, PI3K/Akt, and MAPKs, thereby precipitating transcription‐independent suppression of NF‐*κ*B and AP‐1 within minutes [20]. Moreover, GCs modulate immune cell function through these rapid pathways, suppressing proinflammatory cytokine production (IL‐1*β*, IL‐6, TNF‐*α*, and IFN‐*γ*), inhibiting Th1 and Th17 differentiation, and promoting antiinflammatory mediators such as IL‐10, IL‐1 receptor antagonists, neutral endopeptidases, and lipocortin‐1 [21–23]. Furthermore, these pathways impair antigen‐presenting capacity by inducing a tolerogenic phenotype in dendritic cells (DCs) and limit T‐cell activation through apoptosis or redistribution into lymphoid organs. At the systemic level, GCs transiently elevate circulating neutrophils while reducing their migration to inflammatory sites, further repressing immune activation [24].

However, under chronic stress, persistent GC elevation dysregulates this balance, enhancing NF‐*κ*B‐mediated inflammation and inhibiting anti‐inflammatory cytokines (IL‐4, IL‐19, IL‐13), thereby converting adaptive immune suppression into pathological inflammation [18]. Collectively, these genomic and nongenomic actions provide a sophisticated framework for the dynamic control of immune homeostasis and inflammation resolution.

### 2.3. NF‐*κ*B and Inflammation

NF‐*κ*B is a pleiotropic transcription factor that orchestrates the expression of over 100 genes governing inflammatory and host immune responses. Its activation is triggered by a wide array of stimuli, including pro‐inflammatory cytokines such as IL‐1*β* and TNF‐*α*, pathogen‐associated molecular patterns (PAMPs) like lipopolysaccharides, and other cellular stressors [25]. These stimuli initiate a signaling cascade that culminates in the activation of the I*κ*B kinase (IKK) complex. The IKK complex then phosphorylates inhibitory I*κ*B proteins, targeting them for ubiquitination and subsequent degradation by the 26S proteasome. This degradation liberates NF‐*κ*B dimers, typically heterodimers of the p65 (RelA) and p50 subunits, allowing their translocation into the nucleus. Once in the nucleus, NF‐*κ*B binds to specific *κ*B sites in the promoter regions of target genes to drive a robust proinflammatory gene expression program [26]. This canonical activation pathway is supplemented by a noncanonical pathway mediated by the NF‐*κ*B‐inducing kinase (NIK), ensuring a comprehensive response to diverse stimuli [27].

The NF‐*κ*B/Rel family of proteins shares a conserved Rel homology domain (RHD), which is critical for DNA binding, dimerization, and interaction with I*κ*B proteins [28]. As a central mediator of inflammation, NF‐*κ*B regulates the proliferation, differentiation, and apoptosis of various immune cells and orchestrates the expression of cytokines, chemokines, and adhesion molecules. For instance, NF‐*κ*B activation directs the differentiation of macrophages and guides naïve T cells toward Th1 and Th17 lineages to coordinate the immune response to pathogens [29]. While this inflammatory response is essential for acute host defense, its sustained activation under conditions of chronic stress can lead to prolonged inflammation and tissue damage, contributing to the pathogenesis of chronic inflammatory diseases. Therefore, the precise regulation of NF‐*κ*B activity is crucial for maintaining immune homeostasis [30].

### 2.4. Antagonistic Interaction Between GR and NF‐*κ*B

The intimate involvement of both the GR*α* and NF‐*κ*B in regulating inflammation has positioned their interaction as a critical nexus between the endocrine stress response and the immune system [31]. A primary antiinflammatory function of GR*α* is its ability to antagonize NF‐*κ*B activity through several distinct, yet potentially overlapping, mechanisms [19]. This reciprocal repression is fundamental to immune homeostasis and is a key pathway disrupted by chronic stress.

The most direct mechanism involves a physical, protein–protein interaction between the activated GR*α* and the p65 subunit of NF‐*κ*B. This binding leads to a mutual repression, where GR*α* inhibits NF‐*κ*B’s transcriptional activity, and conversely, NF‐*κ*B can suppress GR*α*‐mediated gene activation [32, 33]. This reciprocal antagonism is dependent on specific domains within each protein, namely the LBD and DBD of GR*α*, and the transactivation domains within the RHD of p65. However, this direct interaction alone may be insufficient for full repression and can require co‐repressor proteins, such as Mad1, to stabilize the complex [34].

A second major mechanism, known as tethering, allows GR*α* to repress NF‐*κ*B‐driven gene expression without directly binding to DNA itself [35]. In this model, the activated GR*α* is recruited to NF‐*κ*B complexes that are already bound to their cognate DNA response elements. This “tethered” GR*α* then interferes with the transcriptional machinery, leading to the potent repression of proinflammatory genes such as *IL-8* and *ICAM-1* [36]. The significance of this pathway is underscored by genome‐wide chromatin immunoprecipitation (ChIP) and sequencing studies, which have identified thousands of potential genomic sites for such tethering interactions and confirmed that GR*α* can repress transcription without displacing NF‐*κ*B from chromatin [37].

Beyond static interactions at gene promoters, GR*α* may also dynamically regulate NF‐*κ*B signaling by influencing its nucleocytoplasmic shuttling. Evidence suggests that activated GR*α* can increase the rate of p65 nuclear export, thereby reducing its nuclear occupancy and transcriptional capacity [36]. This process may involve GR*α*’s interaction with nuclear export machinery or its ability to destabilize the p65 protein by interfering with its interactions with other intranuclear proteins, such as protein kinase A [38, 39]. Collectively, these mechanisms (direct physical interaction, tethering to DNA‐bound complexes, and modulation of nuclear dynamics) form a multilayered system through which GR*α* exerts powerful control over NF‐*κ*B, providing a crucial brake on inflammatory responses.

## 3. Changes in GC Sensitivity: Pathological Modification of GR and GR Resistance

GRs regulate gene expression in nearly all physiological domains of our body, including the immune system, liver metabolism, and nervous system [40]. In stressful situations, GRs play a pivotal role in mediating the effects of GCs to maintain homeostasis. Consequently, changes in the biological sensitivity of GR*α* to GCs can disrupt gene transcription and systemic stress responses, leading to pathological diseases. GC sensitivity is dynamically regulated by multiple factors, including post‐translational modifications (PTMs) of the GR protein, the expression of different GR isoforms, and genetic variations [41, 42]. Chronic stress, in particular, is known to elicit glucocorticoid resistance (GCR), compromising the receptor’s function as a critical antiinflammatory transcription factor. The molecular basis for this altered GC sensitivity involves several key mechanisms, primarily PTMs and the expression of diverse GR isoforms.

### 3.1. GR*α* Phosphorylation

Scores of studies have reported a connection between chronic stress and GR*α* phosphorylation. Stress from chronic isolation in mice was found to decrease the transcriptional activity of GR*α*‐regulated genes by altering the phosphorylation ratio of GR*α* (pGR) at key serine residues (S232 and S246) [43]. In human GR*α*, phosphorylation typically occurs at serine residues 211 and 226, which corresponds to the S246/S232 pGR ratio in mice. The phosphorylation ratio of GR*α* (S211/S226) is a critical determinant of stress‐induced transcriptional activity [44–46].

The overall phosphorylation process of human GR*α* is modulated by kinases at transactivation and cis‐repression domains. For example, p38 mitogen‐activated protein kinase (MAPK) increases GR*α* phosphorylation, which in turn reduces the ligand‐binding sensitivity of GR*α* [47]. Moreover, GR*α* phosphorylation is known to decrease the binding affinity for both GC ligands and target DNA sequences [48]. This impaired binding inhibits the transactivation and cis‐repression of GREs, thereby altering antiinflammatory activity. Furthermore, GR*α* phosphorylation inhibits the physical interaction with the p65 subunit of NF‐*κ*B, leading to the disinhibition and transactivation of NF‐*κ*B‐mediated inflammatory genes [49]. Thus, chronic stress‐induced GR*α* phosphorylation represses GRE‐dependent antiinflammatory gene expression while promoting NF‐*κ*B‐driven inflammation.

### 3.2. GR*α* Acetylation/Deacetylation

Acetylation of proteins, including GR, histone, and chromatin, is another critical PTM that regulates transcriptional activity and inflammatory responses. Human GR*α* are acetylated at lysine 494 and 495 residues within the DBD and deacetylated by histone deacetylase 2 (HDAC2). GR*α* acetylation at these sites hinders its binding to NF‐*κ*B. In contrast, deacetylation of GR*α* by HDAC2 facilitates this protein–protein interaction, enabling the repression of NF‐*κ*B‐induced inflammatory gene transcription [50, 51]. Therefore, GR*α* deacetylation is a key step that allows the receptor to suppress the transcriptional activities of NF‐*κ*B, and inhibition of HDAC2 is associated with decreased GC sensitivity and reduced GR*α*‐mediated suppression of NF‐*κ*B.

### 3.3. GR*α* SUMOylation

Covalent modification of GR by small ubiquitin‐like modifier 1 (SUMO‐1), a process known as SUMOylation, has also been suggested as a regulator of GR function, though its effects remain debated [52]. Three major SUMOylation sites have been identified within the GR*α* protein, located in two regions of the NTD and one within the LBD [53]. Evidence from several studies suggests that conjugation with SUMO‐1 can target GR*α* for proteasomal degradation, thereby diminishing its capacity to interact with and repress NF‐*κ*B signaling. However, SUMOylation can also exert antiinflammatory effects through an alternative pathway. The NF‐*κ*B inhibitor I*κ*B, a key regulator of NF‐*κ*B activation, is itself subject to SUMO modification. Whereas ubiquitination of I*κ*B targets it for proteasomal degradation and thereby permits NF‐*κ*B activation, SUMOylation has the opposite effect, stabilizing I*κ*B and preventing its degradation [54]. Consequently, NF‐*κ*B remains sequestered in the cytoplasm, leading to suppression of its nuclear translocation and transcriptional activity. These observations suggest that a dynamic interplay between ubiquitination and SUMOylation of pivotal signaling proteins such as GR and I*κ*B serves as a fine‐tuning mechanism for NF‐*κ*B‐dependent inflammatory gene expression.

### 3.4. GR Isoform: A Key Determinant of GC Sensitivity

GC sensitivity is largely dictated by the diversity of GR protein isoforms generated from the single human *NR3C1* gene [55]. This diversity, which allows for precise, tissue‐specific tuning of GC responses, arises primarily from two molecular mechanisms: alternative splicing and the use of alternative translation initiation sites. These mechanisms create a complex landscape of GR isoforms, providing multiple avenues for the development of GCR in pathological states [56].

The most well‐characterized source of this diversity is the alternative splicing of exon 9, which generates the two main receptor isoforms, GR*α* and GR*β*. GR*α* is the classic ligand‐binding receptor that mediates the vast majority of known GC effects [57]. In contrast, GR*β* possesses a unique C‐terminus that ablates the ligand‐binding pocket, rendering it incapable of binding GCs. Due to this structural difference, GR*β* can act as an inhibitor of GR*α*, and the cellular GR*α*:GR*β* expression ratio is a critical determinant of GC sensitivity, with an altered ratio being linked to GCR in various inflammatory diseases [58].

A second layer of complexity is introduced by alternative translation initiation from the GR*α* mRNA transcript [57]. At least eight distinct start codons give rise to eight translational isoforms (designated GR*α*‐A through GR*α*‐D3), which feature progressively truncated NTDs [59]. Although all these isoforms bind GCs with similar affinity, their transcriptional capacities differ significantly due to the varying lengths of the N‐terminal activation function‐1 (AF‐1) domain [21]. Generally, the GR*α*‐C isoforms are the most transcriptionally active, while the GR*α*‐D isoforms are the least active [59]. The functional importance of this is strikingly illustrated in DCs. Immature, GC‐resistant DCs predominantly express the less active GR*α*‐D isoforms, whereas upon maturation, they switch to expressing the pro‐apoptotic GR*α*‐A isoform, which confers sensitivity to GC‐induced cell death. This demonstrates how a shift in the translational isoform profile can function as a molecular switch, dramatically altering cellular fate [57].

Finally, inter‐individual variability in baseline GC sensitivity can be attributed to genetic factors, specifically single nucleotide polymorphisms (SNPs) within the *NR3C1* gene. For example, the *ER22/23EK* polymorphism has been associated with relative GCR, whereas the *N363S* polymorphism has been linked to increased GC sensitivity. Certain SNPs can also directly affect isoform expression; the A3669G SNP (rs6198), for instance, stabilizes GR*β* mRNA, leading to a higher GR*β*/GR*α* ratio and contributing to increased resistance [60].

### 3.5. GCR

The mechanisms described above converge to explain how chronic psychological and physiological stress can induce GCR. GCR is frequently observed in individuals undergoing extreme chronic stress, such as parents of children with cancer [61]. This state of reduced cellular sensitivity to GCs is driven by multiple interconnected factors.

A key molecular link involves the co‐chaperone FKBP5. In individuals with post‐traumatic stress disorder, epigenetic demethylation of the *FKBP5* gene leads to its increased expression [62]. Elevated FKBP5 protein then impairs GR*α* signaling by altering the receptor’s chaperone complex, ultimately causing GCR.

A second critical molecular driver of GCR is the expression of the GR*β* splice variant. As a dominant‐negative inhibitor, GR*β* induces GCR by forming transcriptionally inactive GR*α*/GR*β* heterodimers on GREs, which prevents GR*α* homodimers from effectively transactivating target genes [57]. Crucially for the context of this review, GR*β* has been shown to specifically interfere with GR*α*’s ability to repress NF‐*κ*B, thereby promoting a proinflammatory state even in the presence of GCs [63, 64]. The cellular GR*α*:GR*β* ratio thus acts as a rheostat for GC sensitivity [65]. This dynamic can create a vicious feedback loop in inflammatory conditions, as proinflammatory cytokines like TNF‐*α* can selectively increase GR*β* expression, which in turn perpetuates both inflammation and resistance [20].

Functionally, this increased GCR leads to decreased GC sensitivity in immune cells like lymphocytes and neutrophils, resulting in the dysregulated production of proinflammatory cytokines, such as IL‐1*β*, TNF‐*α*, and IL‐6 [66]. In conclusion, chronic stress promotes GCR through a combination of PTMs, GR isoform shifts, and genetic factors. This pathological state impairs the GR*α*’s ability to control inflammation, thereby increasing the risk of inflammatory diseases.

## 4. How GR and NF‐*κ*B Dysfunction Can Elicit Cancer

**Figure 2:**
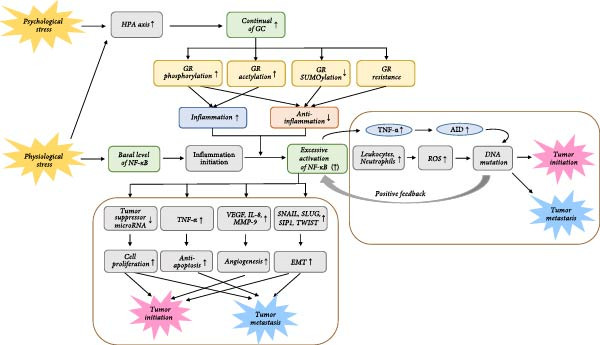
Chronic stress response and cancer‐inducing mechanism of GR and NF‐*κ*B. This map displays how cancer is induced in both the presence and absence of chronic inflammation. Important flows are shown and detailed processes are excluded for concise display. ↑ = activation, increase, ↓ = inactivation, decrease, HPA = hypothalamic–pituitary‐adrenal, GC = glucocorticoid, GR = glucocorticoid receptor, ROS = reactive oxygen species, APC = adenomatous polyposis coli, AID = activation‐induced cytidine deaminase, EMT = epithelial‐mesenchymal transition, XIAP = X‐linked inhibitor of apoptosis, VEGF = vascular endothelial growth factor, iNOS = inducible nitric oxide synthase, COX‐2 = cyclooxygenase‐2, MMP = matrix metalloproteinase.

Chronic exposure to physiological and psychological stress leads to hyperactivation of the HPA axis and overproduction of GCs. The persistent elevation of GCs disrupts the body’s homeostatic balance by inducing alterations in GC sensitivity through mechanisms such as GC resistance and PTMs of the GR. The loss of GR*α*‐mediated inhibition leads to uncontrolled activation of NF‐*κ*B, resulting in chronic low‐grade inflammation. This dysregulated GR–NF‐*κ*B crosstalk transforms the cellular environment into one that is permissive for tumorigenesis, promoting cancer initiation, progression, and metastasis through the altered expression of numerous target genes (Figure 2). A summary of key genes regulated by this antagonistic relationship is presented in Table 1 [14, 67–70].

**Table 1:** Key target genes regulated by the dysregulated antagonistic crosstalk between GR and NF‐*κ*B under chronic stress.

Functional category	Specific gene target	Regulation by GR	Regulation by NF‐*κ*B	Role in stress‐related cancer
Pro‐inflammtory cytokines	IL‐6, TNF‐*α*, IL‐1*β*	Repression (via tethering)	Activation	Promotes a chronic pro‐tumorigenic inflammatory microenvironment
Cell proliferation and cycle	CCND‐1, MYC	Repression (via tethering)	Activation	Drives uncontrolled cell division and oncogenic signaling
Anti‐apoptosis	BCL2, BCL2L1 (Bcl‐xL), XIAP	Repression (via tethering)	Activation	Enables tumor cells to evade programmed cell death, a hallmark of cancer
Angiogenesis	VEGFA, IL‐8	Repression (via tethering)	Activation	Promotes the formation of new blood vessels to supply tumors with nutrients and oxygen
Metastasis and invasion	MMP‐9, ICAM‐1	Repression (via tethering)	Activation	“Degrades the extracellular matrix and facilitates cell adhesion, promoting tumor spread”
Anti‐inflammatory and resolution	GILZ, DUSP‐1 (MKP‐1)	Activation (via GRE)	Indirectly repressed	Suppresses inflammatory kinase signaling and promotes the resolution of inflammation
NF‐*κ*B inhibition (feedback)	NFKBIA (I*κ*B*α*)	Activation (via GRE)	Activation	“Forms a critical negative feedback loop to terminate the NF‐*κ*B signal. GR induction of I*κ*B*α* represents a second, indirect mechanism of NF‐*κ*B inhibition”

*Note:* This table summarizes how the loss of GR‐mediated repression permits the uncontrolled NF‐*κ*B‐driven activation of genes involved in inflammation, proliferation, anti‐apoptosis, angiogenesis, and metastasis, thereby establishing a pro‐tumorigenic microenvironment.

Abbreviatrions: BCL, B‐cell lymphoma; CCND1, cyclin D1; DUSP‐1, dual specificity phosphatase 1; GILZ, glucocorticoid‐induced leucine zipper; ICAM, intercellular adhesion molecule; IL, interleukin; MKP‐1, mitogen‐activated protein kinase phosphatase 1; MMP, matrix metalloproteinase; MYC, myelocytomatosis; NFKBIA, NF‐κB inhibitor alpha; TNF, tumor necrosis factor; VEGFA, vascular endothelial growth factor A; XIAP, X‐linked inhibitor of apoptosis protein.

### 4.1. Mechanisms of NF‐*κ*B‐Driven Carcinogenesis

The sustained activation of NF‐*κ*B, resulting from impaired GR*α* signaling, promotes tumorigenesis through both inflammation‐dependent and inflammation‐independent pathways. Chronic inflammation, orchestrated by NF‐*κ*B, establishes a protumorigenic microenvironment that facilitates cancer development in two primary ways: by enabling immune evasion and by inducing genetic instability [7]. First, tumor cells evade immunosurveillance by recruiting immunosuppressive cells and molecules, such as arginase I and iNOS, which suppress cytotoxic T‐cell responses [71]. NF‐*κ*B further reinforces this immunosuppressive state by directing macrophage polarization toward an M2‐like, low‐tumoricidal phenotype that supports tumor growth [72]. Second, chronic inflammation promotes genetic mutations. Persistent leukocyte and neutrophil infiltration at inflammatory sites generates excessive reactive oxygen species (ROS), causing oxidative DNA damage and triggering a positive feedback loop of further proinflammatory cytokine release and NF‐*κ*B activation [73]. In addition, NF‐*κ*B induces the expression of activation‐induced cytidine deaminase (AID), a mutagenic enzyme that induces somatic mutations in oncogenes and tumor suppressor genes such as c‐MYC and PIM1, thereby accelerating malignant transformation of gastric and hepatic tissues [74, 75].

Beyond inflammatory tumorigenesis, hyperactive NF‐*κ*B directly regulates the expression of genes that are central to the hallmarks of cancer. By modulating tumor suppressor microRNAs and forming a positive feedback loop with the IL‐6/STAT3 pathway, enhanced NF‐*κ*B signaling promotes uncontrolled cell proliferation and confers a potent anti‐apoptotic advantage that allows genetically unstable cells to persist [76]. In mouse models of inflammation‐associated hepatocellular carcinoma, pharmacologic inhibition of NF‐*κ*B restored apoptotic signaling in dysplastic hepatocytes and prevented progression to hepatocellular carcinoma, underscoring its causal role in survival signaling [77]. NF‐*κ*B also promotes angiogenesis by transcriptionally activating pro‐angiogenic mediators, including vascular endothelial growth factor (VEGF), interleukin‐8 (IL‐8), and matrix metalloproteinase‐9 (MMP‐9) [78]. Suppression of NF‐*κ*B in human prostate cancer cells markedly reduced the signaling of key pro‐angiogenic molecules, which inhibited both tumor progression and metastasis [79]. Moreover, NF‐*κ*B drives metastatic dissemination by promoting epithelial–mesenchymal transition (EMT). ChIP assays have identified NF‐*κ*B binding sites in the promoters of genes encoding EMT transcription factors, such as SNAIL, SLUG, and TWIST, whose upregulation facilitates invasion and metastatic spread [80].

Collectively, these mechanisms illustrate the intricate interplay between GR*α* dysfunction and NF‐*κ*B hyperactivation in the progression of cancer. Preclinical models support this bidirectional relationship: GC treatment suppresses peritumoral inflammation and angiogenesis in early‐stage hepatocellular carcinoma, whereas in certain hepatoma cell lines, GCs inhibit apoptosis by maintaining NF‐*κ*B activity. Such findings underscore the context‐dependent duality of GR–NF‐*κ*B signaling in tumor biology.

### 4.2. Functional Genomic Evidence From Human Studies

The molecular model linking chronic stress, GR dysfunction, and NF‐*κ*B‐driven pathology is strongly supported by functional genomic analyses of human tissues. A landmark study on familial caregivers of brain cancer patients, a human model of severe chronic stress, identified a distinct “transcriptional fingerprint” in their peripheral blood monocytes. This signature was characterized by a significant downregulation of genes with GREs and a concurrent upregulation of genes controlled by NF‐*κ*B, providing evidence that psychological stress induces a state of acquired GC resistance and pro‐inflammatory activation at the whole‐genome level [81]. This link has been further solidified at the epigenetic level; a DNA methylation signature derived from human fibroblasts exposed to prolonged physiological stress levels of cortisol was found to be consistently higher in breast cancer tumor samples compared to normal tissue and was associated with more advanced tumor stages [82].

Within the tumor itself, genomic data reveal that the roles of GR and NF‐*κ*B are highly context‐dependent and often paradoxical. While GR activity can serve as a positive prognostic biomarker in malignancies such as adrenocortical carcinoma, it frequently adopts a pro‐tumorigenic function [83]. For instance, transcriptomic data from colorectal cancer (Gene Expression Omnibus (GEO): GSE256159) show that GCs can promote metastasis via a GR‐TET2 interaction [84]. In prostate cancer (GEO: GSE150437, GSE97204), GR*α* is a key driver of acquired resistance to anti‐androgen therapy [85, 86]. This complexity is underscored by single‐cell RNA sequencing of breast cancer cells (GEO: GSE141834), which revealed significant cell‐to‐cell heterogeneity in the GC response, a potential mechanism for therapy evasion [87]. Similarly, while NF‐*κ*B activation often promotes metastasis, as seen in non‐small cell lung cancer, it can also be associated with favorable outcomes. An analysis of metastatic melanoma patients (GEO: GSE145996) found that activating mutations in the NF‐*κ*B inhibitor *NFKBIE* were exclusively present in patients who responded to immunotherapy, suggesting that heightened NF‐*κ*B signaling may prime a more effective anti‐tumor immune response in certain contexts [88]. Mechanistic insights from genome‐wide binding studies further illustrate the dynamic nature of this crosstalk. Foundational ChIP‐seq data from lymphoblastoid cells (GEO: GSE45640) provide global support for the tethering model, in which GR*α* represses NF‐*κ*B activity through protein–protein interaction without displacing it from DNA [89]. However, more recent analyses in lung adenocarcinoma cells reveal a more complex mode of interaction. In this context, co‐activation of GR*α* and the NF‐*κ*B subunit p65 leads to a significant reprograming of their respective cistromes, resulting in their association with novel, shared genomic sites and altering the repertoire of regulated genes in a mutually dependent manner [38]. These findings demonstrate that the GR/NF‐*κ*B interaction is not a static process of simple repression but a dynamic co‐regulation that is highly dependent on the cellular context, reinforcing the complexity of this signaling axis in cancer pathology.

## 5. Discussion

### 5.1. Limitations and Strengths

A primary strength of this work lies in its integrative synthesis, which, to our knowledge, is the first to cohesively connect pathological modifications of the GR under chronic stress to the disinhibition of NF‐*κ*B and the subsequent activation of specific pro‐tumorigenic gene programs. This model is further strengthened by the analysis of public genomic data, providing translational support that bridges preclinical findings with human cancer pathology. However, the most significant limitation is the relative scarcity of clinical studies directly investigating the role of GR PTMs in human cancers. Consequently, our discussion on several PTMs relies on preclinical data, underscoring a critical need for future translational research to validate these mechanisms in patients.

### 5.2. Clinical Implications

While nonpharmacological interventions are beneficial, prolonged chronic stress necessitates pharmacological strategies targeting the dysregulated GR/NF‐*κ*B axis. Current clinical approaches include GCs such as dexamethasone and direct NF‐*κ*B inhibitors such as bortezomib. However, their use is often limited by broad side effects, the development of resistance, and paradoxical protumorigenic roles in certain cancers [90–92]. Therefore, a more sophisticated therapeutic avenue lies in the development of selective GR modulators (SGRMs). These agents are designed to preferentially promote the antiinflammatory functions of GR*α*, such as the inhibition of NF‐*κ*B, while minimizing the transactivation of genes responsible for metabolic side effects, offering the potential for a more precise and safer treatment for stress‐related cancers [93].

## 6. Conclusion

This review synthesizes recent evidence to elucidate how chronic stress promotes carcinogenesis through the bidirectional interaction between the GR and NF‐*κ*B. We have comprehensively detailed how pathological GR modifications, including PTMs and GC resistance, impair GR sensitivity, leading to the disinhibition and hyperactivation of NF‐*κ*B, which in turn drives tumor initiation, proliferation, and metastasis.

Notably, this review provides a unique, integrative analysis that bridges the molecular crosstalk between GR and NF‐*κ*B with the functional consequences of their dysregulation in cancer. By identifying key gene targets at the intersection of these pathways, we offer a mechanistic framework that connects chronic stress signaling directly to the hallmarks of cancer, a synthesis not previously presented in the literature.

However, while the role of GR PTMs in modulating receptor function is clear in preclinical models, there remains a gap in clinical and translational evidence. Therefore, this review underscores the need for future research to characterize the specific PTM signatures of GR in human stress‐related cancers. Elucidating these mechanisms will be critical for developing novel biomarkers and targeted therapeutic strategies that can restore the homeostatic balance of the GR/NF‐*κ*B axis.

**Nomenclature**
ACTH: Adrenocorticotrophic hormone
AID: Activation‐induced cytidine deaminase
ANS: Autonomic nervous system
AP‐1: Activator protein‐1
CAC: Colitis‐associated colon cancer
CBG: Cortisol‐binding globulin
COX‐2: Cyclooxygenase‐2
CREB: cAMP response element‐binding protein
CRH: Corticotrophin‐releasing hormone
CRH‐BP: CRH‐binding protein
DAMPs: Damage‐associated molecular patterns
DBD: DNA binding domain
EPI: Epinephrine
FKBP4: FK506 binding protein 4
GC: Glucocorticoid
GR: Glucocorticoid receptors
GREs: Glucocorticoid response elements
GR*α*: Glucocorticoid receptor alpha
GR*β*: Glucocorticoid receptor beta
HDAC2: Histone deacetylase 2
HPA: Hypothalamic–pituitary–adrenal
IKK: I*κ*B kinase complex
IL‐10: Interleukin 10
iNOS: Inducible nitric oxide synthase
LBD: C‐terminal ligand‐binding domain
MAPK: Mitogen‐activated protein kinase
MMP‐9: Matrix metalloproteinase‐9
NE: Norepinephrine
NF‐*κ*B: Nuclear factor kappa‐light‐chain‐enhancer of activated B cell
NIK: NF‐*κ*B‐inducing kinase
NTD: Disorganized N‐terminal domain
POMC: Proopiomelanocortin
PRRs: Pattern‐recognition receptors
PTM: Post‐translational modifications
RDD: Rel dimerization domain
RHD: Rel homology domain
ROS: Reactive oxygen species
SAM: Sympathetic‐adrenal‐medullary
STAT: Signal transduction and activator of transcription
SUMO‐1: Small ubiquitin‐related modifier‐1
VEGF: Vascular endothelial growth factor.

## Consent

The authors have nothing to report.

## Disclosure

All authors have read and agreed to the manuscript.

## Conflicts of Interest

The authors declare no conflicts of interest.

## Author Contributions

Conceptualization: **Hyun-June Yu and Dae-Hyun Hahm**. Investigation: **Hyun-June Yu**. Resources: **Hi-Joon Park**. Writing – original draft preparation: **Hyun-June Yu**. Write – review and edition: **Bombi Lee and Dae-Hyun Hahm**.

## Funding

This study was supported by National Research Foundation of Korea, RS‐2024‐00409969

## Data Availability

Data are available on request due to privacy/ethical restrictions.
